# Clinical Validation of Four Point‐of‐Care High‐Risk HPV Assays, Including Two Reduced‐Valency Assays, for Cervical Cancer Screening in Low‐Resource Settings

**DOI:** 10.1002/ijc.70534

**Published:** 2026-06-07

**Authors:** Neerja Bhatla, MaryLuz Rol, Seema Singhal, Showket Hussain, Anushree Patil, Pranay Tanwar, Shalini Singh, Vikrant M. Bhor, Kiran Munne, Richa Vashishtha, Shachi Vashist, Eric Lucas, Richard Muwonge, Arianis Tatiana Ramírez, Anju Singh, Vikas Khan, Jyoti Rani, Nasera Firdausi, Sandeep Sisodiya, Rutuja Wakchaure, María Alejandra Picconi, Alejandro Calderón, Margo Bell, Annemie De Smet, Alex Vorsters, Gary M. Clifford, Madhavi Chandra, Jitendra Kumar, Partha Basu

**Affiliations:** ^1^ All India Institute of Medical Sciences New Delhi India; ^2^ Early Detection, Prevention & Infections Branch International Agency for Research on Cancer Lyon France; ^3^ ICMR‐National Institute of Cancer Prevention and Research Noida India; ^4^ ICMR‐National Institute for Research on Women's Health (ICMR‐NIRWoH), Formerly ICMR‐NIRRCH Mumbai India; ^5^ Biotechnology Industry Research Assistance Council Department of Biotechnology New Delhi India; ^6^ Instituto Nacional de Enfermedades Infecciosas – ANLIS Malbrán Buenos Aires Argentina; ^7^ Caja Costarricense de Seguro Social San José Costa Rica; ^8^ Centre for the Evaluation of Vaccination (CEV), Vaccine & Infectious Disease Institute (VAXINFECTIO), Faculty of Medicine and Health Sciences University of Antwerp Antwerp Belgium

**Keywords:** cervical cancer screening, clinical validation, high‐risk HPV assays, low‐resource settings

## Abstract

Persistent infection with high‐risk human papillomavirus (HR‐HPV) is the necessary cause of cervical cancer, with approximately 95% of cases attributable to eight most carcinogenic HPV types (16/18/31/33/35/45/52/58). Several HPV assays have recently been developed in India for use in cervical cancer screening, including reduced‐valency tests targeting seven/eight oncogenic HPV types. We evaluated the clinical accuracy and reproducibility of four such assays: PathoDetect‐HPV‐14, HPV‐Q (14 types), Truenat‐HR‐HPV‐Plus (8 types), and PathoDetect‐HPV‐7 (7 types). Using the VALGENT framework, 1159 cervical samples from the ESTAMPA study conducted in Argentina and Costa Rica were analysed with the new assays. The samples included 97 cases of CIN2+ (19 CIN2, 72 CIN3, and 6 cancers). Assay performance was compared in a blinded manner with established reference tests (Cobas‐4800 and Allplex‐HPV‐HR) and reduced‐valency comparators. Sensitivity, specificity, agreement, and repeatability were assessed. Truenat‐HR‐HPV‐Plus demonstrated a sensitivity of 80.4% (95% CI: 71.1–87.8) and specificity of 91.5% (95% CI: 89.5–93.2) for CIN2+. It met IARC validation criteria and showed non‐inferior performance to Allplex‐HPV‐HR‐8, with relative sensitivity of 1.03 (95% CI: 0.96–1.09) for CIN2+ and 1.00 (95% CI: 0.96–1.04) for CIN3+, and relative specificity of 0.99 (95% CI: 0.97–1.00) and 0.98 (95% CI: 0.97–1.00), respectively. Repeatability was 93.3% (κ = 0.79). PathoDetect‐HPV‐7 showed lower sensitivity (68.1%) with specificity of 89.0%. HPV‐Q and PathoDetect‐HPV‐14 did not meet validation criteria. This study represents the first formal validation of reduced‐valency HPV assays and demonstrates that Truenat‐HR‐HPV‐Plus provides robust clinical performance with higher specificity than 14‐valent assays, supporting its potential to improve screening efficiency and reduce unnecessary referrals.

## What's New?

High‐risk human papillomavirus (hrHPV) testing improves cervical cancer screening by facilitating earlier detection and prevention. In low‐resource settings, however, cost and increased referrals limit its use. At the same time, many new and more affordable assays lack validation. This study evaluated the performance of four reduced‐valency HPV tests, which detect fewer hrHPV genotypes than standard tests, helping improve affordability and accessibility. Analyses show that Truenat‐HR‐HPV‐Plus has strong sensitivity and high specificity, meeting international validation criteria and performing comparably to reference tests. Other evaluated tests were inferior. The findings identify Truenat‐HR‐HPV‐Plus as a reliable, cost‐effective option in low‐ and middle‐income settings.

## Graphical abstract

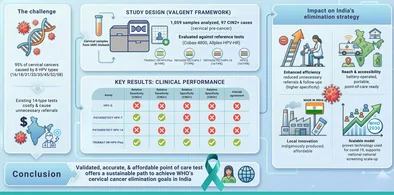

**Abbreviations**
CI: confidence interval
Ct: cycle threshold
HR‐HPV: high‐risk human papillomavirus
LMICs: low‐ and middle‐income countries
NNF: number of women needing follow‐up
NNS: number of women needed to screen
RT: real‐time
WHO: World Health Organization

## 1 Introduction

The World Health Organization (WHO) recommends high‐risk human papillomavirus (HR‐HPV) testing as the primary method for cervical cancer screening [1, 2, 3]. While HPV testing offers clear benefits in terms of sensitivity, it is costly, requires triage, and increases referrals for further management [4]. In low‐ and middle‐income countries like India, affordable point‐of‐care tests with partial genotyping are particularly relevant. While HPV assays are increasingly accessible, understanding of the critical importance of relying on internationally validated tests—such as those meeting the Meijer criteria [5], VALGENT framework [5, 6, 7, 8], and WHO‐TPP guidelines [9]—remains limited.

Evidence suggests that approximately 95% of cervical cancers, and a comparable proportion of CIN3 (cervical intraepithelial neoplasia 3) lesions, are attributable to only eight HR‐HPV types (16/18/31/33/35/45/52/58) with the highest oncogenic potential [3, 10]. In the context of cancer screening, a pragmatic balance must be struck between clinical sensitivity and specificity. Kuhn et al. in South Africa showed that limiting HPV detection to the eight most oncogenic genotypes and adjusting cycle threshold (Ct) cutoffs in the Xpert HPV assay can achieve an optimal balance with sensitivity of 75%–85% and specificity over 90% [11].

As a preparatory step toward implementing a national HPV‐based screening program, we identified four indigenously developed point‐of‐care HPV detection technologies suitable for validation. Notably, two of these assays are designed to detect fewer (7 or 8) while the other two detect 14 HPV genotypes as targeted by most internationally validated commercial tests. Until recently, there were no formal criteria for validating such reduced‐valency HPV tests. In 2024, the International Agency for Research on Cancer (IARC/WHO, France) convened an expert group meeting to refine the validation criteria established by Meijer et al. and the WHO‐TPP guidelines, adapting them to the evaluation of new reduced‐valency HPV tests (henceforth referred to as IARC criteria) [12].

In the current study, four indigenously developed point‐of‐care HPV detection assays with at least partial genotyping for HPV 16 and 18 were evaluated for clinical performance, type‐specific concordance, and inter‐laboratory reproducibility using the VALGENT framework. Two of these tests detecting 14 HPV types were validated using the WHO‐TPP guidelines. The remaining two tests, detecting seven and eight of the most oncogenic HPV types, respectively, were validated according to the newly refined IARC criteria for reduced‐valency tests [12].

## 2 Methods

This study was conducted through a collaboration between the Department of Biotechnology (DBT), Government of India, three Indian academic institutions, namely, the All India Institute of Medical Science (AIIMS) Delhi, Indian Council of Medical Research‐National Institute of Cancer Prevention and Research (ICMR‐NICPR), New Delhi, and ICMR‐National Institute for Research in Reproductive and Child Health (ICMR‐NIRRCH), Mumbai; the University of Antwerp, Centre for the Evaluation of Vaccination, Belgium, and IARC, France.

The indigenous RT‐PCR‐based assays were identified by DBT as potential candidates to be incorporated in the national screening program: HPV‐Q (Genes2Me Pvt. Ltd., Gurugram, India), PathoDetect HPV‐7 and PathoDetect HPV‐14 tests (Mylabs India, Secunderabad, India), and Truenat HR‐HPV‐Plus test (Molbio Diagnostics, Goa, India). Each test was developed by repurposing existing COVID‐19 RT‐PCR platforms. While HPV‐Q and PathoDetect HPV‐14 can detect all 14 HPV types, PathoDetect HPV‐7 only detects HPV types 16/18/31/33/45/52/58. Truenat HR‐HPV‐Plus test includes HPV 35 in addition to the seven types detected with PathoDetect HPV‐7. Three of the tests are capable of detecting HPV 16/18 individually. Truenat HR‐HPV‐Plus test provide a combined result for HPV16/18.

### 2.1 Collection of Cervical Samples

Samples for validation were obtained from the biorepository at IARC. These were collected between 2012 and 2022 as part of the ESTAMPA study, which screened 44,135 women aged 30–64 years in multiple Latin American countries. Clinician‐collected samples in PreservCyt medium were obtained from women who provided written informed consent and were tested locally for HPV using the Cobas‐4800 HPV test and cytology [13]. Leftover aliquots were stored at −70°C. All women who tested positive for HPV and those who were HPV‐negative but had abnormal cytology results were referred for colposcopy. Biopsies were taken from visible lesions. Identification of CIN2 lesions was based on p16 immunostaining. Most histopathology slides (67%) underwent external review, showing 92.5% concordance with local diagnoses.

### 2.2 Sample Processing for Biobanking

Cervical samples along with clinical data from the sites were transferred to IARC biobank for long‐term storage. Informed consent was obtained from all the study participants to biobank their samples and use them for future research. Original samples in Preservcyt were centrifuged, pelleted, and frozen at −70°C at the local laboratories. Samples were shipped to IARC in dry ice.

### 2.3 Selection of Samples for Validation Study

Following the VALGENT protocol we randomly selected 1000 screening samples from the bio‐banked samples collected at two ESTAMPA centers (San Jose, Costa Rica and Buenos Aires, Argentina). This included 772 HPV‐negative and cytology‐negative samples, 59 samples obtained from colposcopy‐negative women not requiring any biopsy, 104 from women with benign biopsies, and 27, 5, and 21 samples were from women with CIN1, CIN2, and CIN3 diagnoses, respectively. Additionally, 71 screening samples with histopathology diagnosis were randomly included (14 CIN2, 51 CIN3, and 6 invasive cancers) to enrich the number of CIN2+ cases.

### 2.4 Processing of Bio‐Banked Samples

At IARC, the pelleted samples were thawed and reconstituted to a final volume of 5.2 mL using PreservCyt solution and subsequently divided into five aliquots. One aliquot was shipped to the lab in Belgium for Allplex HPV‐HR testing. The remaining four aliquots were shipped to the Indian testing laboratories on dry ice. Samples were deidentified and assigned random codes. Laboratory personnel were blinded to cytology, histology, as well as HPV test results from other laboratories.

### 2.5 Analysis of Samples Using Comparator Tests

The Cobas‐4800 (Roche Diagnostics, Switzerland) and Allplex HPV‐HR assays (Seegene Inc., South Korea), both well‐validated assays, were used as comparators.

Cobas‐4800 is an L1 PCR‐based assay that can provide individual results for HPV16 and HPV18, and a grouped result for the other 12 HPV types (HPV 31/33/35/39/45/51/52/56, 58/59/66/68). The test was performed as part of the ESTAMPA study, and we used the test outcomes.

Allplex HPV‐HR is a PCR‐based assay that can individually detect 14 HPV types (HPV 16, 18, 31, 33, 35, 39, 45, 51, 52, 56, 58, 59, 66, 68) [14]. Following an automated DNA extraction using the STARMag Universal Cartridge Kit on the STARlet device (Seegene Inc., South Korea), Allplex testing was performed using CFX96 real‐time (RT) thermocycler (Bio‐Rad, USA).

### 2.6 Analysis of Samples Using the Assays Under Evaluation

Facilities for sample testing using the HPV‐Q (14‐types), PathoDetect (both HPV‐14 and HPV‐7) and Truenat‐HR‐HPV‐Plus‐test (8‐types) were set up at AIIMS, ICMR‐NIRRCH, and ICMR‐NICPR laboratories, respectively. One aliquot of each sample was shipped in dry ice to each of the three Indian study sites. All the sites were blinded to cytology, histology outcomes, and the HPV test results from other laboratories. Technicians were trained by the test manufacturers and were provided with a written standard operating procedure. Subsequent, testing was overseen by the local coordinators without manufacturer involvement. The assay technologies are briefly described below:

#### 2.6.1 HPV‐Q(14)

DNA extraction was carried out using the Genes2Me Rapi X16 DNA Extraction Instrument and its corresponding kits. The assay targets the E6/E7 regions of 14 HPV types DNA. It individually identifies HPV 16 and 18, and the remaining 12 types in a pool and the β‐globin gene as an internal control. Fluorescent dyes are used for detection: HEX (HPV 16), FAM (HPV 18), TEXAS RED (other pooled HR types), and Cy5 (internal control), Ct threshold of ≤ 28 cycles as the positivity criterion. The total run time is approximately 90 min [15].

#### 2.6.2 PathoDetect HPV‐7 and HPV‐14 Tests

RT‐PCR test targets the E6/E7 regions. HPV‐7 targets HPV16/18/31/33/45/52/58. The HPV‐14 assay individually detects HPV 16 and 18 and the remaining 12 HPV types in four groups: HPV 35/59; 39/56/66/68; 31/33/45/52/58; HPV 51 and RNase P gene as internal control. Genomic DNA extraction was conducted in Mylab's Compact XL automated platforms using the Maverick Nucleic Acid Extraction Kit, and genotyping was performed on the benchtop, non‐portable qPCR Real‐Time system, Ct threshold of ≤ 38 cycles as the positivity criterion. Total run time of ~90 min [16].

#### 2.6.3 Truenat HR‐HPV‐Plus Test (8)

RT‐PCR test targets the E7/L1 gene regions of eight HR‐HPV types. The test provides results for HPV 16/18 combined, and a pooled result for types 31/33/35/45/52/58. An exogenous internal control composed of a synthetic DNA construct cloned into a plasmid vector is added to the sample prior to DNA extraction. The control is subsequently co‐extracted and co‐amplified with the target DNA, allowing verification of both extraction efficiency and PCR amplification performance. Genomic DNA extraction, cartridge‐based sample preparation, and genotyping were performed on the Truenat platform. Amplification was carried out for 40 cycles; signals detected within these cycles were classified as positive by the device algorithm. Total run time of ~60 min [17, 18].

### 2.7 Assessing Interlaboratory Reproducibility of the Tests Under Evaluation

Reproducibility analysis was conducted following the VALGENT protocol, using a randomly selected subset of 500 valid specimens from the initial pool of tested samples. To ensure a representative distribution of HR‐HPV‐positive cases, targeting approximately 30% prevalence, 150 HR‐HPV‐positive and 350 HR‐HPV‐negative samples were selected based on results from the Allplex HPV‐HR assay. Each selected sample was first tested in one laboratory using a specific assay, and then the residual aliquots were sent to a second laboratory for blinded testing using the same technology.

Due to the poor initial performance of the HPV‐Q assay, inter‐laboratory reproducibility for this platform was limited to a smaller subset of 100 samples. This set included an equal distribution of HR‐HPV‐positive and negative samples (50 each).

## 3 Statistical Analysis

IARC developed a REDCap interface to facilitate the secure upload of primary export files of each HPV testing batch using randomly assigned codes. Custom R scripts were applied to extract and compile HPV results directly from these files, avoiding manual data entry and reducing the risk of interpretation bias. Epidemiological and clinical data about sample providers were stored in a separate database, inaccessible to the testing laboratories. Statistical analyses were performed using STATA version 17.0 (Stata‐Corp).

The distribution of participant baseline age, HPV test positivity, and final diagnosis was presented as proportions. Histopathological diagnosis, a negative baseline HPV test and cytology, and normal colposcopy determined the final diagnosis.

Data unblinding was done after completion of all tests in presence of study PI (NB) and IARC statistician (RM). The performance of each HPV test for the detection of CIN3+ and CIN2+ was assessed by estimating their sensitivity and specificity. The comparison of the test parameters between Allplex HPV‐HR and Cobas‐4800, and between each indigenous test and Cobas‐4800 and Allplex HPV‐HR were assessed using relative sensitivities, relative specificities and their 95% confidence intervals (CI). The tests with reduced HPV valency (Truenat‐HR‐HPV‐Plus‐test [8‐types] and PathoDetect‐HPV‐7) were evaluated using a comparator that included only the same genotypes detected by the test under evaluation. Accordingly, we conducted a theoretical simulation of evaluation for the Allplex HPV‐HR assay restricted either to eight genotypes (hereafter referred to as “Allplex HPV‐HR‐8,”) or to seven genotypes (hereafter referred to as “Allplex HPV‐HR‐7” included in the candidate tests).

Any HR‐HPV and/or HPV type‐specific concordance between Allplex HPV‐HR and Cobas‐4800, and between each indigenous test and Cobas‐4800, Allplex HPV‐HR, Allplex HPV‐HR‐8, and Allplex HPV‐HR‐7 was presented as proportions and measured by Cohen's kappa statistic. The same statistic was used to evaluate inter‐laboratory reproducibility using the blindly retested 500 randomly selected samples.

### 3.1 Criteria Used for Assay Validation

***For assays detecting 14 HPV types*:** These were validated following Meijer criteria and WHO‐TPP guidelines for validating such tests [5, 9].

***For assays detecting reduced‐valency HPV types* (*less than 14 types*):** For reduced‐valency (< 14 types) HPV tests, validation followed adapted criteria proposed by an expert group convened by IARC. Briefly, the requirements were: (1) meeting WHO‐TPP guidelines for high relative sensitivity (≥ 90% for CIN2+ and ≥ 95% for CIN3+) and specificity (≥ 98% for ≤ CIN), thresholds denoting the lowest bound of the confidence interval, using a validated comparator test; (2) ensuring that comparator tests meet the Meijer criteria, including validated genotyping, and detect at least the genotypes targeted by the reduced valency test; and (3) demonstrating inter‐laboratory reproducibility according to the Meijer criteria (agreement should exceed 87%, and the kappa statistic (κ) should be at least 0.5).

## 4 Results

### 4.1 Characteristics of the Samples Used for Validation

Characteristics of the study population contributing cervical samples are summarized in Table 1. Out of the 1000 randomly selected samples from the screening population, 988 yielded valid results with Allplex HPV‐HR. The 12 samples that yielded invalid results with Allplex HPV‐HR were negative for HPV by Cobas‐4800; these samples were removed from all subsequent analyses. All 71 CIN2+ samples from the enrichment set yielded valid results for the Cobas‐4800 and Allplex HPV‐HR assays. The final validation set consisted of 1059 cervical screening samples.

**TABLE 1:** Distribution of age at screening, HPV test positivity and final diagnosis of women contributing cervical samples for assay validation.

Women characteristics	Type of study population				Total *n* (%)	
	Screening population *n* (%)		Enriched population *n* (%)			
Women assessed	988		71		1059	
Age at screening (in years)						
30–39	362	(36.6)	40	(56.3)	402	(38.0)
40–49	308	(31.2)	24	(33.8)	332	(31.4)
50–64	318	(32.2)	7	(9.9)	325	(30.7)
Cobas‐4800 HPV test results						
Negative	844	(85.4)	5	(7.0)	849	(80.2)
Positive	144	(14.6)	66	(93.0)	210	(19.8)
Final diagnosis						
Normal	935	(94.6)	0	(0.0)	935	(88.3)
CIN1	27	(2.7)	0	(0.0)	27	(2.5)
CIN2	5	(0.5)	14	(19.7)	19	(1.8)
CIN3	21	(2.1)	51	(71.8)	72	(6.8)
Invasive cancer	0	(0.0)	6	(8.5)	6	(0.6)
CIN2+	26	(2.6)	71	(100.0)	97	(9.2)
CIN3+	21	(2.1)	57	(80.3)	78	(7.4)

Abbreviations: CIN, cervical intraepithelial neoplasia; HPV, human papillomavirus.

Mean ages for the screening population (*N* = 988) and the enrichment set (*N* = 71) were 44.8 and 42.5 years, respectively. In the screening population, 14.6% were HPV positive based on the Cobas‐4800 test results, and 2.6% were diagnosed with CIN2+. The enriched set had a 93.0% HPV positivity rate based on the Cobas‐4800 test results, and all cases were histopathology‐confirmed CIN2+ (Table 1).

The HPV positivity rate, considering 14 HPV types for the entire validation set of samples, including screening and enrichment, was 19.8% for Cobas‐4800, 21.1% for Allplex‐HPV‐HR, 24.3% for PathoDetect HPV‐14, and 15.4% for HPV‐Q (Table 2). However, HPV‐Q failed to provide valid results for 50% of the samples. When eight HR‐HPV types were considered, HPV‐positivity was 13.8% for Allplex HPV‐HR‐8 and 15.1% for Truenat HR‐HPV‐8; while with seven HR‐HPV types, HPV‐positivity of 12.8% was reached for Allplex‐HPV‐HR‐7 and 16.3% for PathoDetect‐HPV‐7.

**TABLE 2:** High‐risk HPV Positivity for the different tests evaluated.

HPV tests assessed	Women with valid results^d^	Test positivity *n* (%)	
14 HPV tests^a^			
Cobas‐4800	1059	210	(19.8)
Allplex HPV‐HR	1059	223	(21.1)
PathoDetect HPV‐14	1035	251	(24.3)
HPV‐Q (14)^e^	507	78	(15.4)
8 HR HPV tests^b^			
Allplex HPV‐HR‐8^f^	1059	146	(13.8)
Truenat HR‐HPV‐Plus‐test (8)	1059	160	(15.1)
7 HR HPV tests^c^			
Allplex HPV‐HR‐7^g^	1059	136	(12.8)
PathoDetect HPV‐7	1027	167	(16.3)

Abbreviation: HPV, human papillomavirus.

^a^ Detecting 14 high‐risk HPV types (16, 18, 31, 33, 35, 39, 45, 51, 52, 56, 58, 59, 66, and/or 68).

^b^ Detecting 8 high‐risk HPV types (16, 18, 31, 33, 35, 45, 52, and/or 58).

^c^ Detecting 7 high‐risk HPV types (16, 18, 31, 33, 45, 52, and/or 58).

^d^ All assays evaluated the same number of samples; differences come from invalid results from the given test under evaluation.

^e^ HPV‐Q assay was unable to yield valid results for most samples, significantly limiting the evaluation.

^f^ Test was considered positive only when positive for any of the 8 high‐risk types.

^g^ Test was considered positive only when positive for any of the 7 high‐risk types.

### 4.2 Absolute Sensitivity and Specificity of Reference and Candidate Assays to Detect Any CIN2+

As shown in Table 3, Cobas‐4800 assay showed positive results for any of the 14 HPV types in 89 of 97 CIN2+ cases and negative results in 841 of 962 ≤ CIN1 cases. The absolute clinical sensitivity was 91.8% (95% CI: 84.4–96.4) with a specificity of 87.4% (95% CI: 85.2–89.5). Allplex‐HPV‐HR detected any of the 14 HPV types in 86 CIN2+ cases and 825 in ≤ CIN1 cases, with 88.7% sensitivity (95% CI: 80.6–94.2) and 85.8% specificity (95% CI: 83.4–87.9), comparable to the Cobas‐4800 assay.

**TABLE 3:** Absolute test sensitivity and specificity in detection of any CIN2+ and CIN3+ outcomes.

HPV tests assessed	Women valid results^d^ *n*	Women with outcome *n*	Test TP *n*	Test sensitivity (95% CI) %	Women without outcome *n*	Test TN *n*	Test specificity (95% CI) %
*CIN2+ outcome*							
14 HPV tests^a^							
Cobas‐4800	1059	97	89	91.8 (84.4–96.4)	962	841	87.4 (85.2–89.5)
Allplex‐ HPV‐HR	1059	97	86	88.7 (80.6–94.2)	962	825	85.8 (83.4–87.9)
PathoDetect HPV‐14	1035	97	76	78.4 (68.8–86.1)	938	763	81.3 (78.7–83.8)
HPV‐Q (14)	507	56	31	55.4 (41.5–68.7)	451	404	89.6 (86.4–92.2)
8 HR HPV tests^b^							
Allplex HPV‐HR‐8	1059	97	76	78.4 (68.8–86.1)	962	892	92.7 (90.9–94.3)
Truenat‐HR‐HPV‐Plus‐test (8)	1059	97	78	80.4 (71.1–87.8)	962	880	91.5 (89.5–93.2)
7 HR HPV tests^c^							
Allplex HPV‐HR‐7	1059	97	72	74.2 (64.3–82.6)	962	898	93.3 (91.6–94.8)
PathoDetect‐HPV‐7	1027	94	64	68.1 (57.7–77.3)	933	830	89.0 (86.8–90.9)
*CIN3+ outcome*							
14 HPV tests							
Cobas‐4800	1059	78	71	91.0 (82.4–96.3)	981	842	85.8 (83.5–88.0)
Allplex HPV‐HR	1059	78	69	88.5 (79.2–94.6)	981	827	84.3 (81.9–86.5)
PathoDetect HPV‐14	1035	78	64	82.1 (71.7–89.8)	957	770	80.5 (77.8–82.9)
HPV‐Q (14)	507	42	21	50.0 (34.2–65.8)	465	408	87.7 (84.4–90.6)
8 HR HPV tests^b^							
Allplex HPV‐HR‐8	1059	78	63	80.8 (70.3–88.8)	981	898	91.5 (89.6–93.2)
Truenat HR‐HPV‐Plus‐test (8)	1059	78	63	80.8 (70.3–88.8)	981	884	90.1 (88.1–91.9)
7 HR HPV tests ^c^							
Allplex HPV‐HR‐7	1059	78	59	75.6 (64.6–84.7)	981	904	92.2 (90.3–93.8)
PathoDetect HPV‐7	1027	77	56	72.7 (61.4–82.3)	950	839	88.3 (86.1–90.3)

Abbreviations: CI, confidence interval; CIN, cervical intraepithelial neoplasia; HPV, human papillomavirus; TP, true positives; TN, true negatives.

^a^ Detecting 14 high‐risk HPV types (16, 18, 31, 33, 35, 39, 45, 51, 52, 56, 58, 59, 66, and/or 68).

^b^ Detecting 8 high‐risk HPV types (16, 18, 31, 33, 35, 45, 52, and/or 58).

^c^ Detecting 7 high‐risk HPV types (16, 18, 31, 33, 45, 52, and/or 58).

^d^ All assays evaluated the same number of samples; differences come from invalid results from the given test under evaluation.

The PathoDetect HPV‐14 assay reported positive HPV results for 76 of 97 CIN2+ cases and reported negative results for 763 of 938 ≤ CIN1 samples from 1035 valid samples, leading to a clinical sensitivity of 78.4% (95% CI: 68.8–86.1) and a specificity of 81.3% (95% CI: 78.7–83.8).

The HPV‐Q test had limited clinical relevance, producing valid results for only 507 out of 1059 samples. Within this constrained subset, it exhibited a clinical sensitivity for CIN2+ detection of 55.4% (95% CI: 41.0–68.7) and a specificity of 89.6% (95% CI: 86.4–92.2).

Truenat HR‐HPV‐Plus‐test, targeting eight HR‐HPV types, yielded valid results for all 1059 samples. It detected HR‐HPV in 78 CIN2+ cases and tested HR‐HPV negative in 880 ≤ CIN1 cases, demonstrating a clinical sensitivity of 80.4% (95% CI: 71.1–87.8) and specificity of 91.5% (95% CI: 89.5–93.2).

The PathoDetect HPV‐7 assay, targeting seven HR‐HPV types, provided valid results for 1027 samples. It detected HR‐HPV in 64 of 94 CIN2+ cases and was negative for 830 of 933 ≤ CIN1 samples, resulting in a clinical sensitivity of 68.1% (95% CI: 57.7%–77.3%) and a clinical specificity of 89.0% (95% CI: 86.8%–90.9%), as shown in Table 3.

### 4.3 Absolute Sensitivity and Specificity of Reference and Candidate Tests to Detect Any CIN3+

Table 3 shows that the Cobas‐4800 assay detected CIN3+ lesions with 91.0% (95% CI: 82.4–96.3) clinical sensitivity and 85.8% (95% CI: 83.5–88.0) specificity. Overall, the Allplex‐HPV‐HR assay performed similarly, with a sensitivity of 88.5% (95% CI: 79.2–94.6) and a specificity of 84.3% (95% CI: 81.9–86.5).

PathoDetect HPV‐14 assay demonstrated 82.1% sensitivity (95% CI: 71.7–89.9) and 80.5% specificity (95% CI: 77.8–82.9), identifying 64 of 78 CIN3+ cases and reporting negative results for 770 of 957 ≤ CIN2 cases. HPV‐Q (14) assay, which targets 14 HPV types, demonstrated a clinical sensitivity of 50.0% (95% CI: 34.2–65.8) for CIN3+ and a specificity of 87.7% (95% CI: 84.4–90.6).

Truenat HR‐HPV‐Plus‐test, detected 63 of 78 CIN3+ cases and was negative for 884 of 981 ≤ CIN2 cases, yielding a clinical sensitivity of 80.8% (95% CI: 70.3–88.8) and specificity of 90.1% (95% CI: 88.1–91.9), as shown in Table 3. It is worth noting that out of the six screen‐detected cancers included in the validation, Truenat‐HR‐HPV‐Plus‐test missed only one associated with HPV39, a type not detected by this 8‐type assay.

The PathoDetect HPV‐7 assay demonstrated a sensitivity of 72.7% (95% CI: 61.4–82.3) and a specificity of 88.3% (95% CI: 86.1–90.3) for CIN3+ detection, as shown in Table 3.

### 4.4 Relative Sensitivity, Specificity, and Concordance of Candidate Tests Compared to Reference Tests, Including Reduced‐Valency Comparators

Allplex HPV‐HR showed non‐inferiority compared to the Cobas‐4800 assay for both CIN2+ and CIN3+ detection, with relative sensitivity values of 0.97 (95% CI: 0.92–1.02) and 0.97 (95% CI: 0.92–1.03), respectively and relative specificity values of 0.98 (95% CI: 0.96–1.00) for both endpoints, fulfilling the recommendations in the WHO‐TPP guidelines. The overall agreement between the Allplex HPV‐HR and Cobas‐4800 tests for detecting 14 HPV types was 93.9% (kappa 0.81) (Table S1).

Table 4 presents the relative sensitivity and specificity for the assays under evaluation compared to Allplex HPV‐HR, as well as the reduced‐valency simulation for Allplex HPV‐HR‐8 and ‐7, and the Cobas‐4800 assays for HPV16/18. Concordance values are shown in Table S1.

**TABLE 4:** Relative test sensitivity and specificity in detection of CIN2+ and CIN3+ outcomes.

Test assessed	Comparator test	Relative test sensitivity (95% CI)	Relative test specificity (95% CI)
*CIN2+ outcome*			
Allplex HPV‐HR^a^	Cobas‐4800^a^	0.97 (0.92–1.02)	0.98 (0.96–1.00)
PathoDetect HPV‐14^a^	Cobas‐4800^a^	0.85 (0.76–0.95)	0.93 (0.90–0.96)
	Allplex‐HR‐HPV^a^	0.88 (0.79–0.97)	0.95 (0.92–0.98)
HPV‐Q(14)^a^	Cobas‐4800^a^	0.57 (0.34–0.80)	1.04 (1.00–1.08)
	Allplex‐HPV‐HR^a^	0.60 (0.37–0.82)	1.07 (1.03–1.11)
Truenat HR‐HPV‐Plus‐test(8)^b^	Allplex‐HPV‐HR‐8^b^	1.03 (0.96–1.09)	0.99 (0.97–1.00)
PathoDetect HPV‐7^c^	Allplex‐HPV‐HR‐7^c^	0.93 (0.82–1.03)	0.95 (0.93–0.98)
*CIN3+ outcome*			
Allplex HPV‐HR^a^	Cobas‐4800^a^	0.97 (0.92–1.03)	0.98 (0.96–1.00)
PathoDetect HPV‐14^a^	Cobas‐4800^a^	0.90 (0.81–0.99)	0.94 (0.91–0.97)
	Allplex‐HPV‐HR^a^	0.93 (0.84–1.02)	0.96 (0.92–0.99)
HPV‐Q(14)^a^	Cobas‐4800^a^	0.53 (0.23–0.82)	1.05 (1.01–1.10)
	Allplex‐HPV‐HR^a^	0.54 (0.25–0.83)	1.08 (1.03–1.12)
Truenat HR‐HPV‐Plus‐test(8)^b^	Allplex‐HPV‐HR‐8^b^	1.00 (0.96–1.04)	0.98 (0.97–1.00)
PathoDetect HPV‐7^c^	Allplex‐HPV‐HR‐7^c^	0.97 (0.86–1.07)	0.96 (0.94–0.98)

Abbreviations: CI, confidence interval; CIN, cervical intraepithelial neoplasia; HPV, human papillomavirus.

^a^ Detecting 14 high‐risk HPV types (16, 18, 31, 33, 35, 39, 45, 51, 52, 56, 58, 59, 66, and/or 68).

^b^ Detecting 8 high‐risk HPV types (16, 18, 31, 33, 35, 45, 52, and/or 58).

^c^ Detecting 7 high‐risk HPV types (16, 18, 31, 33, 45, 52, and/or 58).

Compared with Allplex HPV‐HR, the PathoDetect HPV‐14 assay had a relative sensitivity of 0.88 (95% CI: 0.79–0.97) for CIN2+ and 0.93 (95% CI: 0.84–1.02) for CIN3+ and a relative specificity of 0.95 (95% CI: 0.92–0.98) for CIN2+ and 0.96 (95% CI: 0.92–0.99) for CIN3+. The concordance between PathoDetect HPV‐14 and Allplex HPV‐HR was 82.6% (kappa 0.51) for detecting any oncogenic HPV and 98.1% (kappa 0.79) for detecting HPV 16/18.

HPV‐Q assay, among the subset with valid data, showed poor clinical performance compared to Allplex HPV‐HR, with relative sensitivity of 0.60 (95% CI: 0.37–0.82) and 0.54 (95% CI: 0.25–0.83) for detecting CIN2+ and CIN3+, respectively.

When compared to Allplex HPV‐HR‐8, Truenat HR‐HPV‐Plus‐test achieved relative sensitivity values of 1.03 (95% CI: 0.96–1.09) for CIN2+ and 1.00 (95% CI: 0.96–1.04) for CIN3+, and relative specificity values of 0.99 (95% CI: 0.97–1.00) for CIN2+ and 0.98 (95% CI: 0.97–1.00) for CIN3+, fulfilling the IARC criteria for validation. The assay showed strong concordance with Allplex‐HPV‐HR‐8 for the detection of the eight HR‐HPV types (agreement: 95.7%, kappa: 0.82). Agreement for HPV 16 and 18 detection was even higher, at 97.5% (kappa: 0.77).

The PathoDetect HPV‐7 assay, when compared to Allplex HPV‐HR‐7, showed a relative sensitivity of 0.93 (95% CI: 0.82–1.03) for CIN2+ and 0.97 (95% CI: 0.86–1.07) for CIN3+, and a relative specificity of 0.95 (95% CI: 0.93–0.98) for CIN2+ and 0.96 (95% CI: 0.94–0.98) for CIN3+. The overall agreement with Allplex HPV‐HR‐7 for the seven high‐risk types was 89.6% (Cohen's kappa: 0.58), while agreement for HPV 16 and/or 18 was 92.9% (kappa: 0.50).

### 4.5 Assessment of Interlaboratory Reproducibility of the Tests Under Validation

The interlaboratory reproducibility data for the different assays are summarized in Table 5. Only the Truenat HR‐HPV‐Plus test showed substantial reproducibility among all the tests, meeting the standards set in the IARC validation criteria. The overall interlaboratory agreement for HR‐HPV detection with the Truenat HR‐HPV‐Plus‐test was 93.3% (kappa 0.79; 95% CI: 0.70–0.88). The interlaboratory agreement for PathoDetect HPV‐14 was 63.6% (kappa 0.29) and that for PathoDetect HPV‐7 was 80.1% (kappa 0.52). The interlaboratory agreement for the valid samples for HPV‐Q was 37.2% (kappa 0.01).

**TABLE 5:** Interlaboratory agreement of the different HPV tests.

Test assessed	HPV type	Women assessed *n*	Test results combination				Agreement (95% CI) %	Kappa (95% CI)
			Positive, positive *n*	Positive, negative *n*	Negative, positive *n*	Negative, negative *n*		
PathoDetect HPV‐14	16, 18, 31, 33, 35, 39, 45, 51, 52, 56, 58, 59, 66, and/or 68	434	99	24	134	177	63.6 (59.1–68.1)	0.29 (0.21–0.38)
	16 and/or 18	434	33	12	20	369	92.6 (90.2–95.1)	0.63 (0.54–0.73)
	16	434	29	7	19	379	94.0 (91.8–96.2)	0.66 (0.57–0.75)
	18	434	4	7	10	413	96.1 (94.3–97.9)	0.30 (0.21–0.39)
	31, 33, 35, 39, 45, 51, 52, 56, 58, 59, 66, and/or 68	434	78	27	144	185	60.6 (56.0–65.2)	0.22 (0.14–0.30)
HPV‐Q (14)	16, 18, 31, 33, 35, 39, 45, 51, 52, 56, 58, 59, 66, and/or 68	43	11	2	25	5	37.2 (22.8–51.7)	0.01 (−0.15–0.17)
	16 and/or 18	43	2	4	0	37	90.7 (82.0–99.4)	0.46 (0.21–0.71)
	16	43	1	4	0	38	90.7 (82.0–99.4)	0.31 (0.09–0.52)
	18	43	1	0	1	41	97.7 (93.2–102.2)	0.66 (0.38–0.94)
	31, 33, 35, 39, 45, 51, 52, 56, 58, 59, 66, and/or 68	43	7	1	29	6	30.2 (16.5–44.0)	0.02 (−0.10–0.14)
Truenat HR‐HPV‐Plus test (8)	16, 18, 31, 33, 35, 45, 52, and/or 58	481	79	27	5	370	93.3 (91.1–95.6)	0.79 (0.70–0.88)
	16 and/or 18	481	42	16	4	419	95.8 (94.1–97.6)	0.78 (0.70–0.87)
	31, 33, 35, 45, 52, and/or 58	481	43	17	4	417	95.6 (93.8–97.5)	0.78 (0.69–0.87)
PathoDetect HPV‐7	16, 18, 31, 33, 45, 52, and/or 58	413	75	19	63	256	80.1 (76.3–84.0)	0.52 (0.42–0.61)
	16 and/or 18	413	39	24	15	335	90.6 (87.7–93.4)	0.61 (0.52–0.71)
	16	413	29	13	22	349	91.5 (88.8–94.2)	0.58 (0.48–0.67)
	18	413	10	20	7	376	93.5 (91.1–95.8)	0.39 (0.30–0.49)
	31, 33, 45, 52, and/or 58	413	33	9	66	305	81.8 (78.1–85.6)	0.38 (0.29–0.46)

Abbreviations: CI: confidence interval; HPV: human papillomavirus.

## 5 Discussion

This study comprehensively evaluated the clinical performance and interlaboratory reproducibility of four indigenously developed Indian RT‐PCR‐based HPV assays, including two reduced‐valency tests, specifically designed for low‐resource settings. The findings demonstrate the importance of rigorous validation before assays are used in programmatic settings or prescribed to women. This work provides the first formal validation of LMIC‐adapted assays targeting only seven or eight HR‐HPV types, offering a realistic assessment of their clinical utility.

Neither 14‐valency HPV assay here evaluated met validation criteria. HPV‐Q yielded valid results for only half the samples, with suboptimal clinical performance and reproducibility, making it unsuitable for clinical use. PathoDetect‐HPV‐14 likewise failed to achieve required sensitivity for CIN2+/CIN3+ detection and showed poor reproducibility (κ = 0.30).

The PathoDetect‐HPV‐7 assay, targeting seven HR‐HPV types included in the nonavalent HPV vaccine, did not meet IARC reduced‐valency criteria, including detection of at least the eight most carcinogenic HPV types. Still, despite limited sensitivity (72.7%), it showed high specificity (88.3%) for CIN3+, exceeding Allplex‐HPV‐HR (84.3%), and Cobas‐4800 (85.8%). Its limited reproducibility remains a critical limitation that can be improved upon.

Conversely, the Truenat HR‐HPV‐Plus test met all IARC non‐inferiority and interlaboratory reproducibility criteria, with relative sensitivities of 1.03 (95% CI: 0.96–1.09) for CIN2+ and 1.00 (95% CI: 0.96–1.04) for CIN3+, and relative specificity of 0.98 (95% CI: 0.97–1.00). Its markedly higher specificity for CIN2+ (92.7%) and CIN3+ (91.5%) compared to Allplex‐HPV‐HR (85.5%, 84.3%) and Cobas‐4800 (87.4%, 85.8%) underscores its potential to minimize unnecessary referrals in screening programs.

The Truenat platform for Truenat HR‐HPV‐Plus test is well‐suited to resource‐limited settings, offering portability, minimal training requirements, battery operation, room‐temperature cartridge storage, and multiplex capability. Its successful deployment in primary health centers and district hospitals across India supported scaling up of Covid testing in India during the pandemic [19]. The same platforms may be used to scale up national HPV screening, though the two‐cartridge processing limit may restrict use of the technology in high‐volume settings. IARC is currently conducting an implementation research study in Tamil Nadu, India, assessing the Truenat HR‐HPV‐Plus test in a rural primary health center, evaluating its field feasibility and efficacy [20]. The outcomes will inform scale‐up decisions and provide a model for other LMICs.

Screening assays with high sensitivity but low specificity, such as 14‐valent HPV assays, pose challenges in LMICs with high HPV prevalence, often exacerbated by high HIV prevalence, where elevated test positivity rates and inadequate capacity to triage or treat HPV‐positive women can make screening programs unsustainable. As demonstrated by Kuhn et al. in South Africa, restricting detection to the eight most oncogenic genotypes and optimizing cycle threshold (Ct) cutoffs can substantially improve specificity without major loss of sensitivity, optimizing referral for triage and offering a pragmatic balance for sustainable screening. This approach enables improved clinical management through more efficient identification of women at risk and a reduction in unnecessary referrals. Although a slight decrease in sensitivity for CIN2+ detection may occur, the higher screening coverage achieved through improved efficiency is expected to compensate for missed cases. This forms the rationale for the use of reduced‐valency tests [12].

In a population (Sweden) with comprehensive cervical screening coverage and follow ups, absolute impact has been calculated according to HPV type by estimating “number of women needed to screen” (NNS) and “number of women needing follow‐up” (NNF) to detect/prevent one case of cervical cancer [21]. Ranking of HPV types by NNS and NNF was consistent with the seven to eight types included in the PathoDetect‐HPV‐7 and Truenat HR‐HPV‐Plus, and screening for HPV types of lower oncogenic potential (HPV35,39,51,56,59,66,68) was significantly less efficient. The authors recommended screening tests to consider restricting the HPV types to improve screening efficiency, even in a high‐income setting.

When interpreting absolute CIN2+ and CIN3+ sensitivity of reduced valency tests, of the type presented here, it should be remembered that not all CIN2, and not even all CIN3, progress to cervical cancer, and that the risk of progression is known to be HPV type dependent [22]. Cross‐sectional meta‐analyses have shown that the importance of HPV16, but also HPV18 and HPV45, increases between CIN2 and even CIN3 to cervical cancer, while the importance of other HR‐HPV types proportionally decreases. Whereas approximately 95% of cervical cancer is attributable to HPV16/18/31/33/45/52/58, this fraction is more like 85% for CIN3 and 70% for CIN2 [23]. This means that in our study where CIN2+ includes predominantly CIN3 and CIN2 and only very few cancers, a 100% type‐specific sensitivity against HPV16/18/31/33/45/52/58‐positive CIN2+, although it equates with an approximate 80% absolute sensitivity for any CIN2+, can be expected to be associated with a higher absolute sensitivity (> 90%) against any cervical cancer.

The key limitation of the study is that all the tests were performed in highly controlled settings and even the best performing test may have implementation challenges (e.g., stability of test kits in different temperatures, variability between different lots of kits) when put to real‐world use In addition, the platforms are not validated using self‐sampling approach which is ongoing and essentially requires future high‐throughput screening scale‐up capacity. Finally, a limitation is that the Truenat HR‐HPV‐Plus test currently lacks an internal control to verify sample adequacy, which should be added, as it will be important given the variability in sample collection expected during large‐scale implementation.

## 6 Conclusion

This study provides a scalable, resource‐efficient model for HPV assay validation in LMICs, combining national and international expertise. India now has a validated point‐of‐care HPV assay. Before nationwide rollout, a scale‐up phase with rigorous monitoring of all indicators is essential to ensure consistent performance across diverse contexts, healthcare providers, and operational conditions, including expanded production and distribution.

## Author Contributions

**Neerja Bhatla:** conceptualization, funding acquisition, methodology, project administration, supervision, writing – original draft, writing – review and editing. **MaryLuz Rol:** conceptualization, methodology, project administration, supervision, validation, writing – original draft, writing – review and editing. **Seema Singhal:** investigation, writing – review and editing. **Showket Hussain:** investigation, project administration, writing – review and editing. **Anushree Patil:** investigation, project administration, writing – review and editing. **Pranay Tanwar:** investigation, writing – review and editing. **Shalini Singh:** investigation, writing – review and editing. **Vikrant M. Bhor:** investigation, writing – review and editing. **Kiran Munne:** investigation, writing – review and editing. **Richa Vashishtha:** investigation, writing – review and editing. **Shachi Vashist:** investigation, writing – review and editing. **Eric Lucas:** data curation, formal analysis, software, validation, writing – review and editing. **Richard Muwonge:** conceptualization, data curation, formal analysis, methodology, software, validation, writing – review and editing. **Arianis Tatiana Ramírez:** methodology, writing – original draft, writing – review and editing. **Anju Singh:** investigation, writing – review and editing. **Vikas Khan:** investigation, writing – review and editing. **Jyoti Rani:** investigation, writing – review and editing. **Nasera Firdausi:** investigation, writing – review and editing. **Sandeep Sisodiya:** investigation, writing – review and editing. **Rutuja Wakchaure:** investigation, writing – review and editing. **María Alejandra Picconi:** investigation, writing – review and editing. **Alejandro Calderón:** investigation, writing – review and editing. **Margo Bell:** investigation, writing – review and editing. **Annemie De Smet:** investigation, writing – review and editing. **Alex Vorsters:** methodology, writing – review and editing. **Gary M. Clifford:** methodology, writing – review and editing. **Madhavi Chandra:** investigation, writing – review and editing. **Jitendra Kumar:** methodology, writing – review and editing. **Partha Basu:** conceptualization, funding acquisition, methodology, supervision, writing – original draft, writing – review and editing.

## Funding

Funded by DBT–BIRAC under the Grand Challenges India initiative and supported by the Gates Foundation (INV‐061732). MB is supported by a predoctoral fellowship of the Research Foundation—Flanders (FWO), Belgium (11PJK24).

## Ethics Statement

Ethical approvals were obtained from the IARC Ethics Committee, the Institute Ethics Committee All India Institute of Medical Sciences, and all participating institutions. All participants had consented to biobanking of their samples.

## Conflicts of Interest

A.V. is cofounder and minority shareholder of a spin‐off company of the University of Antwerp (Sampl.id) that produces a first‐void urine collection device. A.V. holds patents related to this work. The University of Antwerp obtained project grants, honoraria fee for lectures, presentations, speaker bureaus or other financial support from Becton Dickinson, MSD, Roche, GSK, Hologic, Merck, Abbott, Cepheid, and Seegene Inc. All funds are handled and managed by the University of Antwerp. All other authors declare no competing interests.

## Supporting information


**Table S1:** HPV type‐specific agreement between the different tests.

## Data Availability

The data that support the findings of this study are available from the corresponding author upon reasonable request.
