# Supplementary material for: Clinical Validation of Four Point‐of‐Care High‐Risk HPV Assays, Including Two Reduced‐Valency Assays, for Cervical Cancer Screening in Low‐Resource Settings
Source: Int J Cancer. 2026 Jun 7;159(8):2004–14. doi: 10.1002/ijc.70534 (PMC13495801; doi:10.1002/ijc.70534)
Supplement: Supplementary file 1 [file IJC-159-2004-s001.pdf]

Supplementary Material for

Clinical validation of four point-of-care high-risk HPV assays, including two reduced-valency assays, for cervical cancer screening in low-resource settings

Neerja Bhatla, MaryLuz Rol, Seema Singhal, Showket Hussain, Anushree Patil, Pranay Tanwar, Shalini Singh, Vikrant M Bhor, Kiran Munne, Richa Vashishtha, Shachi Vashist, Eric Lucas, Richard Muwonge, Arianis Tatiana Ramírez, Anju Singh, Vikas Khan, Jyoti Rani, Nasera Firdausi, Sandeep Sisodiya, Rutuja Wakchaure, María Alejandra Picconi, Alejandro Calderón, Margo Bell, Annemie De Smet, Alex Vorsters, Gary M Clifford, Madhavi Chandra, Jitendra Kumar, Partha Basu

Table of Contents

Supplementary table 1: HPV type-specific agreement between the different tests

Supplementary table 1: HPV type-specific agreement between the different tests

Test 1	Test 2	HPV type	Women assessed	Test results combination				Agreement (95% CI)	Kappa (95% CI)
				Test 1+ Test 2+	Test 1+ Test 2-	Test 1- Test 2+	Test 1- Test 2-		
			n	n	n	n	n	%	
Allplex-HPV-HR	Cobas-4800	16, 18, 31, 33, 35, 39, 45, 51, 52, 56, 58, 59, 66, and/or 68	1059	184	39	26	810	93.9 (92.4 - 95.3)	0.81 (0.75 - 0.87)
		16 and/or 18	1059	48	1	14	996	98.6 (97.9 - 99.3)	0.86 (0.80 - 0.92)
		16	1059	33	1	11	1014	98.9 (98.2 - 99.5)	0.84 (0.78 - 0.90)
		18	1059	15	0	3	1041	99.7 (99.4 - 100.0)	0.91 (0.85 - 0.97)
		31, 33, 35, 39, 45, 51, 52, 56, 58, 59, 66, and/or 68	1059	143	41	18	857	94.4 (93.0 - 95.8)	0.80 (0.74 - 0.86)
PathoDetect-HPV-14	Allplex-HPV-HR	16, 18, 31, 33, 35, 39, 45, 51, 52, 56, 58, 59, 66, and/or 68	1035	146	75	105	709	82.6 (80.3 - 84.9)	0.51 (0.45 - 0.57)
		16 and/or 18	1035	40	9	11	975	98.1 (97.2 - 98.9)	0.79 (0.73 - 0.85)
		16	1035	29	5	11	990	98.5 (97.7 - 99.2)	0.78 (0.72 - 0.84)
		18	1035	11	4	4	1016	99.2 (98.7 - 99.8)	0.73 (0.67 - 0.79)
		31, 33, 35, 39, 45, 51, 52, 56, 58, 59, 66, and/or 68	1035	113	69	119	734	81.8 (79.5 - 84.2)	0.43 (0.37 - 0.49)
	Cobas-4800	16, 18, 31, 33, 35, 39, 45, 51, 52, 56, 58, 59, 66, and/or 68	1035	146	64	105	720	83.7 (81.4 - 85.9)	0.53 (0.47 - 0.59)
		16 and/or 18	1035	42	20	9	964	97.2 (96.2 - 98.2)	0.73 (0.67 - 0.79)
		16	1035	31	13	9	982	97.9 (97.0 - 98.8)	0.73 (0.67 - 0.79)
		18	1035	11	7	4	1013	98.9 (98.3 - 99.6)	0.66 (0.60 - 0.72)
		31, 33, 35, 39, 45, 51, 52, 56, 58, 59, 66, and/or 68	1035	109	52	123	751	83.1 (80.8 - 85.4)	0.45 (0.40 - 0.51)
HPV-Q(14)	Allplex-HPV-HR	16, 18, 31, 33, 35, 39, 45, 51, 52, 56, 58, 59, 66, and/or 68	507	55	70	23	359	81.7 (78.3 - 85.0)	0.43 (0.35 - 0.52)
		16 and/or 18	507	12	22	17	456	92.3 (90.0 - 94.6)	0.34 (0.25 - 0.43)
		16	507	10	14	18	465	93.7 (91.6 - 95.8)	0.35 (0.26 - 0.44)
		18	507	1	9	0	497	98.2 (97.1 - 99.4)	0.18 (0.13 - 0.23)
		31, 33, 35, 39, 45, 51, 52, 56, 58, 59, 66, and/or 68	507	36	64	14	393	84.6 (81.5 - 87.8)	0.40 (0.32 - 0.48)

	Cobas-4800	16, 18, 31, 33, 35, 39, 45, 51, 52, 56, 58, 59, 66, and/or 68	507	53	65	25	364	82.2	(78.9 -	85.6)	0.44	(0.35 -	0.52)
		16 and/or 18	507	12	29	17	449	90.9	(88.4 -	93.4)	0.30	(0.21 -	0.38)
		16	507	10	19	18	460	92.7	(90.4 -	95.0)	0.31	(0.23 -	0.40)
		18	507	1	11	0	495	97.8	(96.6 -	99.1)	0.15	(0.10 -	0.20)
		31, 33, 35, 39, 45, 51, 52, 56, 58, 59, 66, and/or 68	507	34	53	16	404	86.4	(83.4 -	89.4)	0.42	(0.34 -	0.51)
Truenat-HR-HPV-Plus-test(8)	Allplex-HPV-HR-8	16, 18, 31, 33, 35, 45, 52, and/or 58	1059	130	16	30	883	95.7	(94.4 -	96.9)	0.82	(0.76 -	0.88)
		16 and/or 18	1059	46	3	23	987	97.5	(96.6 -	98.5)	0.77	(0.71 -	0.83)
		31, 33, 35, 45, 52, and/or 58	1059	90	14	13	942	97.5	(96.5 -	98.4)	0.86	(0.80 -	0.92)
	Cobas-4800	16 and/or 18	1059	47	15	22	975	96.5	(95.4 -	97.6)	0.70	(0.64 -	0.76)
PathoDetect-HPV-7	Allplex-HPV-HP-7	16, 18, 31, 33, 45, 52, and/or 58	1027	96	36	71	824	89.6	(87.7 -	91.4)	0.58	(0.52 -	0.64)
		16 and/or 18	1027	41	7	66	913	92.9	(91.3 -	94.5)	0.50	(0.44 -	0.55)
		16	1027	31	2	28	966	97.1	(96.0 -	98.1)	0.66	(0.60 -	0.72)
		18	1027	10	5	54	958	94.3	(92.8 -	95.7)	0.24	(0.19 -	0.28)
		31, 33, 45, 52, and/or 58	1027	57	34	25	911	94.3	(92.8 -	95.7)	0.63	(0.57 -	0.69)
	Cobas-4800	16 and/or 18	1027	45	16	62	904	92.4	(90.8 -	94.0)	0.50	(0.44 -	0.56)
		16	1027	35	8	24	960	96.9	(95.8 -	97.9)	0.67	(0.61 -	0.73)
		18	1027	10	8	54	955	94.0	(92.5 -	95.4)	0.22	(0.17 -	0.27)

HPV: human papillomavirus; CI: confidence interval
